# PubFocus: semantic MEDLINE/PubMed citations analytics through integration of controlled biomedical dictionaries and ranking algorithm

**DOI:** 10.1186/1471-2105-7-424

**Published:** 2006-10-02

**Authors:** Maksim V Plikus, Zina Zhang, Cheng-Ming Chuong

**Affiliations:** 1 Department of Pathology, Keck School of Medicine, University of Southern California, Los Angeles, California, USA; 2 Department of Medicine, David Geffen School of Medicine, University of California, Los Angeles, Los Angeles, California, USA

## Abstract

**Background:**

Understanding research activity within any given biomedical field is important. Search outputs generated by MEDLINE/PubMed are not well classified and require lengthy manual citation analysis. Automation of citation analytics can be very useful and timesaving for both novices and experts.

**Results:**

PubFocus web server automates analysis of MEDLINE/PubMed search queries by enriching them with two widely used human factor-based bibliometric indicators of publication quality: journal impact factor and volume of forward references. In addition to providing basic volumetric statistics, PubFocus also prioritizes citations and evaluates authors' impact on the field of search. PubFocus also analyses presence and occurrence of biomedical key terms within citations by utilizing controlled vocabularies.

**Conclusion:**

We have developed citations' prioritisation algorithm based on journal impact factor, forward referencing volume, referencing dynamics, and author's contribution level. It can be applied either to the primary set of PubMed search results or to the subsets of these results identified through key terms from controlled biomedical vocabularies and ontologies. NCI (National Cancer Institute) thesaurus and MGD (Mouse Genome Database) mammalian gene orthology have been implemented for key terms analytics. PubFocus provides a scalable platform for the integration of multiple available ontology databases. PubFocus analytics can be adapted for input sources of biomedical citations other than PubMed.

## Background

PubMed (MEDLINE) is the leading online public database of biomedical literature records [1]. It is an essential tool used by scientists and physicians to monitor research developments in any given field of science or medicine. While it is simple and easy to use, PubMed provides only a limited set of data mining tools. One can narrow searches in PubMed by successive queries with multiple criteria, such as year, organism, author, geographic location, journal etc. Such search narrowing strategies can require advanced query construction skills and are not intuitive. In addition, search narrowing decisions have to be largely based on pre-existing knowledge (or best guess) of the user. For example, when conducting a search the user has to know ahead of time authors of interest or the time period when crucial developments have been published. As a result, it is often difficult to comprehend the state of research activity without manually sorting articles based on their titles and abstracts.

Several publicly available citation post-processing tools have been developed to compensate for the lack of computational capabilities of PubMed. Services such as SLIM [2], MedKit [3] and PubMed Assistant [4] provide more intuitive search interfaces, encouraging users to take advantage of available PubMed search filters in refining their data mining strategy. Other services, such as XplorMed [5,6], PubFinder [7], Vivisimo^® ^[8] and GoPubMed [9] match PubMed search results with additional databases to classify articles into relevant topics based on ontology. XplorMed extracts keywords from the abstracts of the PubMed hits by analysis of word co-occurrence. Subsequent classification and selection are based on those keywords. To establish ontology, PubFinder uses an independently generated occurrence frequency database of 100,000 terms extracted from abstracts available through PubMed since 1990. GoPubMed uses the Gene Ontology database [10], including over 19,000 terms organized in three sub-ontologies: cellular location, molecular function or biological process. However, most of these services make a use of only one filtering mechanism or of only one biomedical database.

Many other useful biomedical databases have not been integrated into PubMed analysis. For example, as of the August 2006 BioMed Central provides references to as many as 2,704 biomedical databases. Many of these databases are textual, containing unique collections of terms that can be included in citations' analytics [11]. All of these databases can potentially become a useful addendum in citation analytics.

Despite a growing number of tools for textual processing of PubMed citations, there has been little done on developing efficient statistical representation tools of major bibliometric data. PubMed lacks simple statistical analyses of relevant search records, and the user cannot obtain a quick introduction to the topic of interest through publication trends, publications with the most impact, and names of leading researchers in the field. An alternative to PubMed, Web of Science^® ^(The Thomson Corporation) is a subscription-based service that provides only basic analysis of search results by parameters such as publication year, author's name, and source title on up to 2,000 records at a time [12]. However, statistical outputs generated by Web of Science^® ^are purely volumetric and do not provide any impact ranking of publications or authors.

Ranking of publications and authors can be based on "human factor" bibliometric parameters such as journal impact factor and volumetric data on forward referencing, both of which are used as common numeric indicators of a publication's impact.

1) Journal impact factor (IF) determines the average significance and quality of information in any given article published in a respective journal. Impact factor is calculated as follow [13,14]:

**IF **= All citations in **YEAR **to articles in **JOURNAL **during (**YEAR-1**) + (**YEAR-2**)/All articles in **JOURNAL **during (**YEAR-1**) + (**YEAR-2**)

Journals with a higher impact factor are generally harder to get published in. These journals have a more stringent and rigorous review process and more strict requirements as to the quality of the presented data. If a manuscript is not accepted in a journal with a higher rank, it will ultimately be attempted in an alternative journal of lower rank (one with a lower impact factor). In most instances, higher quality data will be published in journals with a higher impact factor. While this is not universally true and evidence exists against this statement [15], "*there is nothing better and it (impact factor) has the advantage of already being in existence and is, therefore, a good technique for scientific evaluation. Experience has shown that in each specialty the best journals are those in which it is most difficult to have an article accepted, and these are the journals that have a high impact factor*" [16].

2) The volume of the forward citations and the citation trend over the lifetime of an article reflects its acceptance by the scientific community. Thus, it is obvious that if published findings represent a significant milestone in the field, they will be heavily cited.

Unlike other bibliometric parameters (such as chronological and volumetric parameters), both numerical parameters described above are dependent on a human factor. Unfortunately, PubMed does not account for them. The SIGAPS software accounts for journal impact factor [17]. This software serves the purpose of generating graphic reports on a researcher's publication activity and tags each relevant article with the journal impact factor value. For each researcher, it breaks down all his/her articles into arbitrary groups based on the values of the impact factor (from high to low). SIGAPS shows the proportion of articles published in sources with high impact factors versus those with low impact factors.

Further development of ranking utility accounting for both impact factor and forward citations among other factors is required. Taken separately, impact factor and volume of forward citations can be misleading. Leading journals within a narrow biomedical field can have a low impact factor that does not adequately represent the publication's impact. Also, self-referencing is a common practice among some scientists, and articles with apparently high forward citation level might have few citations from other scientists. Therefore, it will be desirable to develop a numeric index based on both parameters.

Integration of the variety of available biomedical ontology databases and both major "human factor"-based bibliometric parameters (impact factor and volume of the forward citations) into efficient free-source semantic citations analytics tool has not been done. We have developed PubFocus to compensate for this apparent lack of an important functionality.

## Implementation

**Figure 1:**
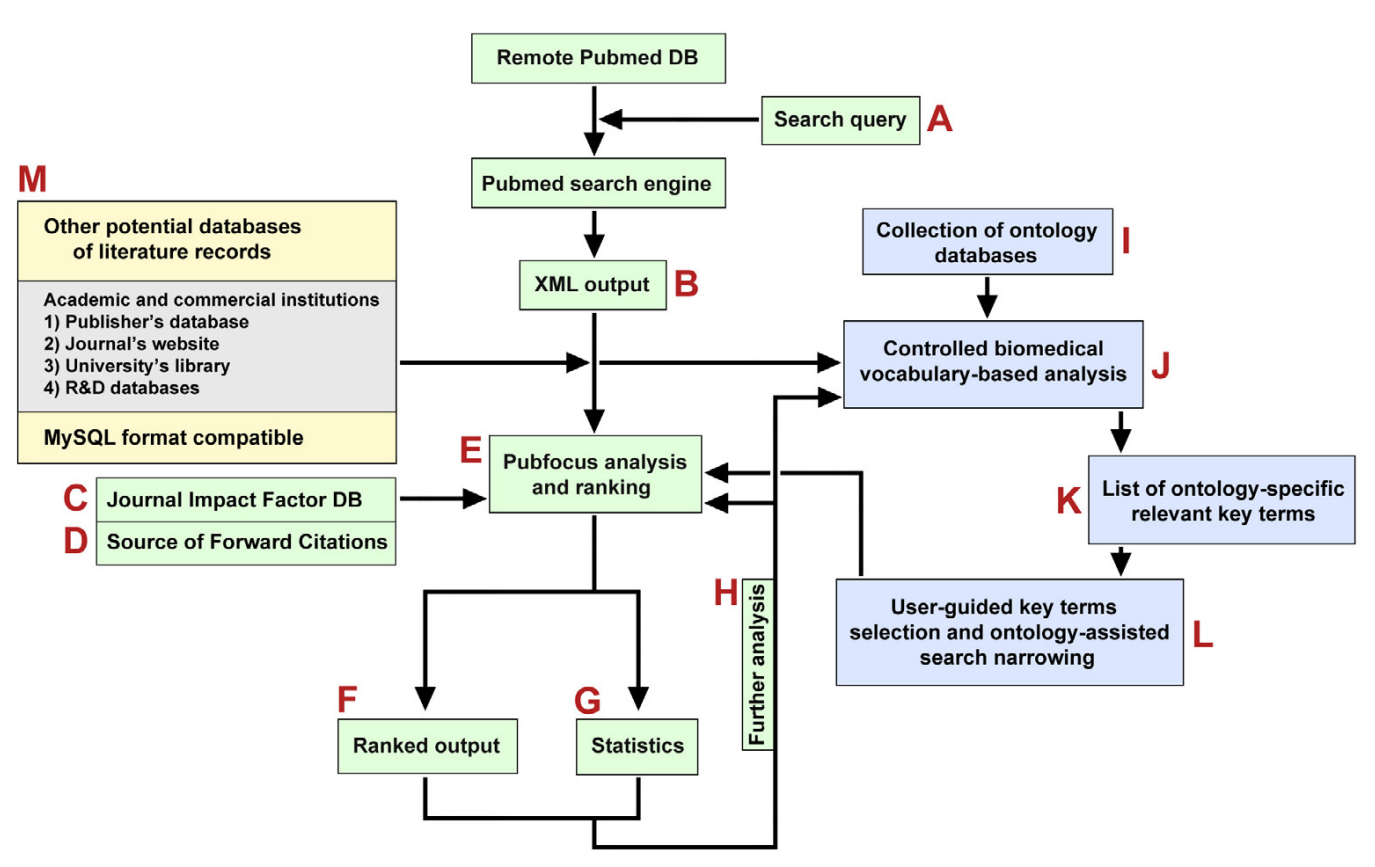
**Flow-chart of PubFocus functionality**. **A) **The initial PubFocus search is preformed by using several complementing search menus: basic and advanced menus with various search limits for each, and detailed, allowing experienced users to construct their own search queries. **B) **Data on relevant search matches is extracted from PubMed in packets of 50 XML-formatted records. **C) **Impact factor is assigned to each PubMed record from the locally hosted journal ranking database. **D) **Number of forward citations is established from one of three alternative sources: PubMed Basic, PubMed Central or Google™ Scholar and is assigned to each PubMed record. **E) **Local temporary database of relevant PubMed records enriched with impact factor and volumetric data on forward citations is created on PubFocus server. **F) **Publications can be sorted and viewed ranked by either: publication date, first author, last author, impact factor, forward citations or PIF (aka Combined Impact Factor; Fig. 2G). Sorting can be done either in ascending or descending modes. **G) **Alternatively, statistical analysis on relevant publications can be performed. PubFocus sorts publications by various parameters and provides ranking tables with semi-graphical output. **H) **Through the use of intuitive limiting tools user can create a focused search query and concentrate statistical analysis on just a subset of initial search results without reinitiating often lengthy data acquisition process. **I) **Multiple biomedical databases, such as ontologies can be integrated with PubFocus using standard MySQL format. **J) **MySQL full-text search allows automatic search and extraction of matching terms from titles and abstracts of relevant citations. **K) **Relevant terms are sorted either based on their occurrence rate or by the build-in ontology categories. **L) **Matching terms are presented in form of semi-graphical output, allowing selection and search narrowing procedures.

**Figure 2:**
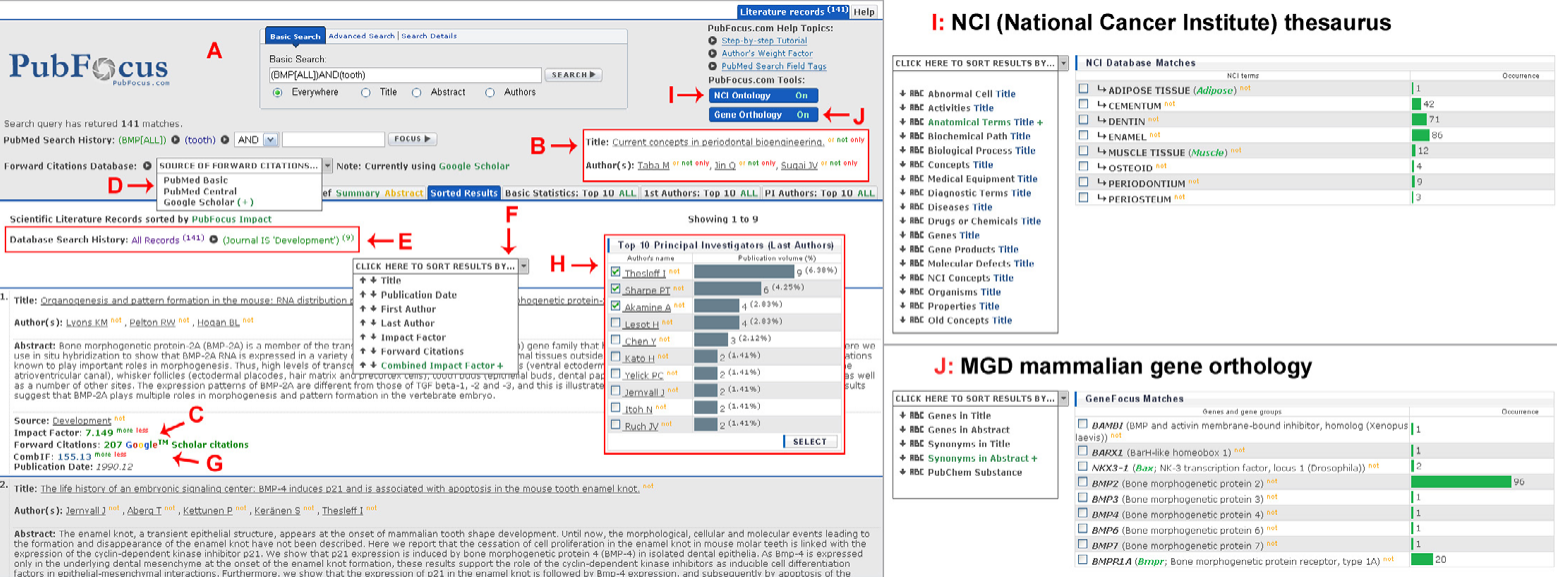
**Screenshot demonstrating PubFocus functional elements**. **A) **PubFocus search menu containing basic, advanced and detailed sub-menus. **B) **Hyperlink-based search limiting tools allow interactive search output navigation. Four hyperlinks are commonly provided with AND, OR, NOT and ONLY operators. **C) **Corresponding impact factor (IF) of the journal and a number of forward citations are shown for each record. **D) **Drop-down menu allows selection of forward citations source to be used in further analysis: PubMed Basic, PubMed Central or Google™ Scholar. **E) **Interactive navigation tool allows quick back and forth focusing on the subsets of the initial search using temporary local database. **F) **Drop-down menu allows viewing of search results sorted by either: publication date, first author, last author, impact factor, forward citations or PIF (aka Combined Impact Factor). Sorting can be done either in ascending or descending modes. **G) **Combined Impact Factor is calculated for each record based on Publication Impact Factor (see Implementation). **H) **Ranking tables provide semi-graphical output of the search results categorized by various bibliometric parameters (see Implementation section). Ranking tables contain limiting tools (hyperlinks, check-boxes) allowing selection and analysis of various subsets of the initial search records. **I) **"NCI Ontology" tool outputs terms from NCI thesaurus occurring within titles and/or abstracts of the relevant citations. Terms are presented in semi-graphical form. It includes search limiting tools (hyperlinks, check-boxes). **J) **"Gene Orthology" tool outputs terms from MGD mammalian gene orthology database occurring within titles and/or abstracts of the relevant citations. Terms are also presented in semi-graphical output including search limiting tools (hyperlinks, check-boxes).

The initial PubFocus search is preformed by using several complementing search menus: basic and advanced menus with various search limits for each, and detailed, allowing experienced users to construct their own search queries (Fig. 1A; Fig. 2A). Syntax of the search queries is identical to that of PubMed. Previously generated PubMed queries can be imported into PubFocus by simply copying the entire query string into the search field under the detailed menu. Initial search results are arranged chronologically (20 records are displayed at a time) and can be viewed in three alternative modes: brief, summary and abstract. In each mode both the title and authors' names are hyperlinked to allow interactive search output navigation. Four hyperlinks are commonly provided for each author's name (AND, OR, NOT and ONLY), allowing quick focus on search subsets including or excluding any particular author, as well as simple link-out to all publications by any given author (Fig. 2B). Additionally, for each record an impact factor (IF) of the journal and a number of forward citations are obtained by interfacing parallel databases (Fig. 2C). Impact factor is obtained from a locally hosted and manually built journal ranking database, which includes current impact factors for 7,525 unique source titles (based on the 2005 edition of Journal Citation Report^® ^by The Thomson Corporation). Volumetric data on forward citations is obtained through automated parsing of HTML outputs for the individual PubMed Central (PMC) records (commonly known as "cited in PMC" data) and matching Google™ Scholar records. This information can be used to judge the rank of the publication, as a higher impact factor and higher volume of forward referencing would indicate seniority of the article.

PubFocus provides a set of statistical tools for bibliometric analysis of relevant records. In the first step, data on relevant records is extracted from the PubMed server in packets of 50 XML-formatted records (up to a total of 2500 records per analysis) and written into a local temporary database (Fig. 1B, 1E). Remote extraction of the XML-formatted records was chosen over loading the entire PubMed database (approximately 31.6 – 46.3 GB in size [18]) into the local relational database in order to maintain lightweightness and ease of transferability of the application between servers and to avoid necessity for frequent updates and maintenance. In addition, this method excludes a need for developing a separate search engine. Here, relevance of citations to the search query is determined by the PubMed search engine. In the second step, the local database is enriched with IFs and volumetric data on forward citations (Fig. 1C, 1D; Fig. 2C). The user can choose to collect data on forward citations from three alternative sources: PubMed Basic, PubMed Central or Google™ Scholar (Fig. 2D). Data acquisition from PubMed Basic is fast, yet it only allows establishing the presence or absence of forward references within the PubMed Central database (i.e. "yes or no" mode). Alternatively, both PubMed Central and Google™ Scholar provide numeric values of forward citations, but data harvesting from these sources can be lengthy and is recommended for smaller sets of records. In general, Google™ Scholar provides higher values of forward citations for the same publication than PubMed Central does. In the recent formal study forward citation data from Google™ Scholar showed a substantial degree of overlap with that from proprietary Web of Science^® ^[19]. While forward citations appear in the Google™ Scholar database earlier in the lifecycle of the publication than in PubMed Central database, Google™ Scholar "returns a smaller number of citing references" than Web of Science^® ^but "provides a large set of unique citing material" [19]. While "it is clear that Google™ Scholar provides unique citing material", "the exact composition of this citing material should also be more thoroughly examined so that scholars will have a clear idea what is and is not included in Google™ Scholar searches".

Statistical analysis is performed in the second step. Basic statistics employ a simple volumetric approach (similar to the analysis done by Web of Science^®^) to compute, sort, and provide semi-graphical output of the results (Fig. 1G). Basic statistics can be viewed by accessing the "Basic Statistics" tab. Analysis includes that of publication trends over the years, top publishing first authors (commonly scientists with most contribution to the paper), top publishing last authors (commonly principal investigators), top fields of research, top research topics, top publication sources based on volume or impact factor, publication types, and publication languages. In addition, search-narrowing tools allow selection and display of subsets of relevant records matching any of the above listed parameters. For example, one can select to display records published by top three principal investigators or records published in certain journals only (such as a small subset of "high-profile" articles published in journals like *Nature *or *Science *with high impact factors). Back and forth focusing on the subsets of the initial search is done using a temporary local database without additional time-consuming external data harvesting (Fig. 1G → 1H → 1E → 1G; Fig. 2E).

We have also developed means for the integration and use of biomedical databases in the assisted citations' analytics (Fig. 1I). We have designed a standard MySQL format and integrated MySQL full-text search to allow automatic extraction of matching terms from titles and abstracts of relevant citations (Fig. 1J). These terms are sorted either based on their occurrence rate or by build-in ontology categories. Matching terms are presented in the form of semi-graphical output, allowing similar selection and search narrowing procedures as throughout the rest of the PubFocus portal (Fig. 1K, 1L). While multiple databases qualified for the integration, we chose NCI (National Cancer Institute) thesaurus and MGD (Mouse Genome Database) mammalian gene orthology (Fig. 2I, 2J). Both of these databases are large and can be useful for a wide scientific audience rather than for small interest groups only. NCI thesaurus represents a "major effort to integrate molecular and clinical cancer-related information within a unified biomedical informatics framework, with controlled terminology as its foundational layer". It includes some 49,000 biomedical concepts and 146,000 synonyms "separated into 20 logically distinct *kinds*" including anatomical terms, diagnostic terms, diseases, drugs and chemicals etc. [20,21]. NCI thesaurus has not been previously integrated into any PubMed citations analytics applications. MGD mammalian gene orthology includes around 65,500 symbols and names and an additional 24,000 synonyms of mammalian genes. It is one of the most comprehensive and inclusive mammalian gene databases [22].

Additional statistics employ custom algorithms to provide more accurate ranking of the search results. Particularly, PubFocus uses several determinants of publications' and authors' impact to automatically attempt to identify most prominent publications and authors whose papers can be considered significant within the given field of search.

1) Combined Impact Factor (CombIF) is calculated as CombIF = IF*citations-over-age index to account for:

a) Impact factor (IF) of the publication source.

b) Age of the publication and presence of forward references in either one of three databases mentioned above (**citations-over-age index**). Calculation of **citations-over-age index **is performed based either on Table 1 (if PubMed Basic mode is used) or Table 2 (if either PubMed Central or Google™ Scholar mode is used). In general, **citations-over-age index **boosts the IF value of new and cited articles proportionally to the number of forward citations and reduces the IF value of old articles that have not been cited ("dead-end" articles).

**Table 1:** Algorithm of citations-over-age index determination in PubMed Basic mode

		**Age**			
		
		< = 1 year	> 1 and < = 3 years	>3 and < = 5 years	> 5 years
**Forward Citations**	No forward citations	**1**	**1**	**0.75**	**0.5**
	Present forward citations	**1.5**	**1.25**	**1**	**1**

**Table 2:** Algorithm of citations-over-age index determination in either PubMed Central or Google™ Scholar mode

		**Age**			
		
		< = 1 year	>1 and < = 3 years	> 3 and < = 5 years	> 5 years
**Forward Citations**	No forward citations	**1**	**1**	**0.75**	**0.5**
	Present forward citations	1+(0.4*n)	1+(0.2*n)	1+(0.1*n)	1+(0.1*n)

where **n **= number of forward citations

2) Cumulative impact factor (CIF) is calculated as **CIF = ∑(IF) **and represents the cumulative value of a journal's IF where any given author has been published.

3) Author's Rank (AR) was introduced to make important adjustments to AIF. It is calculated as **AR = ∑(CombIF/contribution index) **to account for:

a) Combined Impact Factor (CombIF, see above).

b) The role of author in the publication (**contribution index**). Contribution index of the first and last authors (the most contributing authors) on the paper is set at **1**, keeping CombIF value unchanged. Contribution level of the middle authors demonstrates high degree of variability [23]. Often, however, contribution of the second author is greater than contribution of the remaining middle authors [24]. Therefore, the **contribution index **of second authors is set at **1/2 **and for remaining middle authors it is set at **1/3, **diluting the initial CombIF value. While an alternative strategy could have been implemented where total **contribution indices **of all authors of a paper should add to "1", this approach was dismissed because papers with large authors' lists will produce contributions diluted to an insignificant extent.

Ranked results can be viewed in the form of sorted citation records by accessing the "Sorted Results" tab (Fig. 1F; Fig. 2F). Processed publications can be sorted by publication date, first author, last author, impact factor, forward citations or CombIF (Fig. 2G). Sorting can be done either in ascending or descending modes. Sorting by CombIF in descending mode is the most informative, as it outputs publications with the highest established impact first. In addition, by accessing the "Basic Statistics" tab the user can view distribution of either impact factors, volume of forward citations or CombIFs among all publications within the database.

By accessing the "1st Authors" and "PI Authors" tabs the user can access ranking tables of both first and last authors within the given field of search (Fig. 2H). Ranking is provided based on both CIF and AR (see above). Two sets of tables are generated. The first set accounts for the publications where respective authors are either first or last in the list of authors only. The second set accounts for all publications including those where respective authors are not the primary contributors (i.e. middle authors). Ranking tables by AR on all publications (last table on the page) is the most informative as it ranks authors based on their cumulative established impact within the field of search.

Upon completion of data acquisition for any given search query, the user can quickly browse between "Sorted Results", "Basic Statistics", "1st Authors", "PI Authors" "NCI Thesaurus" and "Gene Orthology" tabs without reinitiating the often lengthy data acquisition process (Fig. 2E). Until a new search is performed, statistical analysis is done by accessing data stored in the temporary local database.

## Results

### Comparison of available sorting functionalities between: PubFocus, PubMed, Web of Science^® ^and Google™ Scholar (Fig. 3)

**Figure 3:**
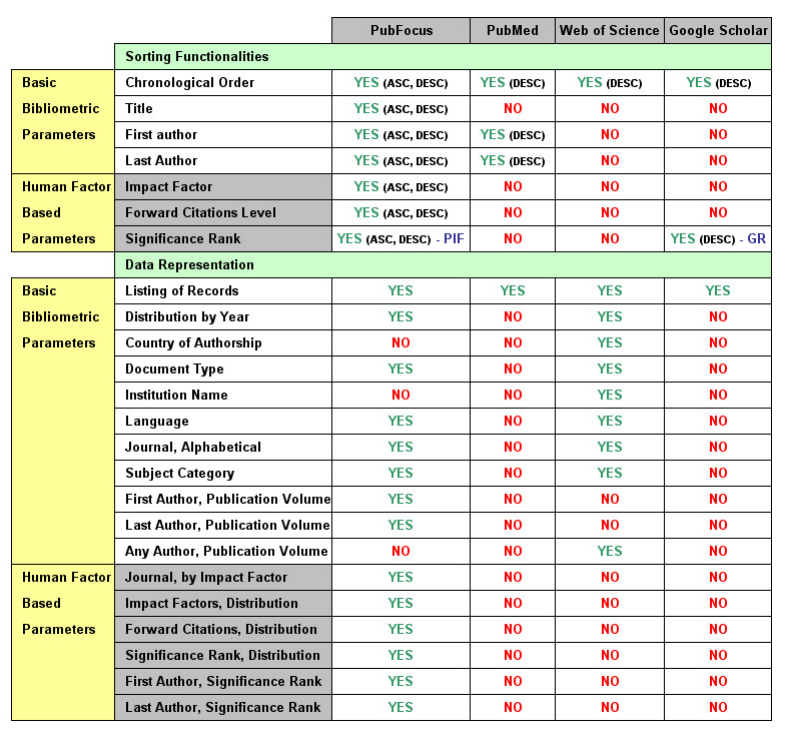
**Comparison of available sorting and data representation functionalities**. Comparison of available sorting and data representation functionalities between PubFocus, PubMed, Web of Science^® ^and Google™ Scholar. In comparison with other services PubFocus provides various data sorting modes based on basic bibliometric as well as human factor-based parameters (impact ranking). Likewise, PubFocus contain various data representation modes including both sorted listings and ranking tables. Unlike Web of Science^® ^that provides ranking tables based on basic bibliometric parameters only, PubFocus generates ranking tables that sort publication sources and authors based on their estimated impact (see Implementation).

By default, both PubMed and Web of Science^® ^output search results in chronological order with most recent records at the top. In Web of Science^®^, this sorting method is enforced and cannot be changed. In PubMed, the user has an option to alternatively sort records in descending order by first author, last author or journal title. Google™ Scholar sorts search results by internal Google™ rank by default, with presumably most relevant records displayed at the top. Alternatively, Google™ Scholar can sort results in descending chronological order. Unlike these services, PubFocus allows both descending and ascending sorting of the search results by either publication date, first author, last author, impact factor, forward citations or CombIF (Fig. 2G; Fig. 3). This provides users with the freedom to arrange articles according to their requirements. For example, in order to view the articles with most impact, users would sort by CombIF in descending mode. In order to view most recent articles, users would sort by publication date. In order to view articles published in top journals first, users would sort by impact factor in descending mode.

### Comparison of PubFocus ranking versus Google™ Scholar and Scirus™ ranking

Google™ Scholar and Scirus™ are two popular biomedical citations services that provide ranking of the search results according to their impact on the field. Google™ Scholar uses a proprietary undisclosed ranking algorithm that takes into account "the full text of each article as well as the article's author, the publication in which the article appeared and how often it has been cited in scholarly literature" [25]. However, concerns exist regarding the accuracy of the Google™ Scholar ranking system because of the inherent lack of accuracy of forward citations and poor definition of the scholarly nature of the sources within which the search is performed [19]. Scirus™ relevance ranking is based on two parameters: "location and frequency of a search term within a result" and "number of links to a page" [26]. The relevancy of search results to the search query in PubFocus derives directly from PubMed. PubFocus ranking of relevant results is calculated based on CombIF and accounts for the impact factor of the publication source, volume of forward citations in the PubMed Central or Google™ Scholar databases, and age of the publication (the age of the publication determines forward referencing dynamics over time; see Implementation).

**Figure 4:**
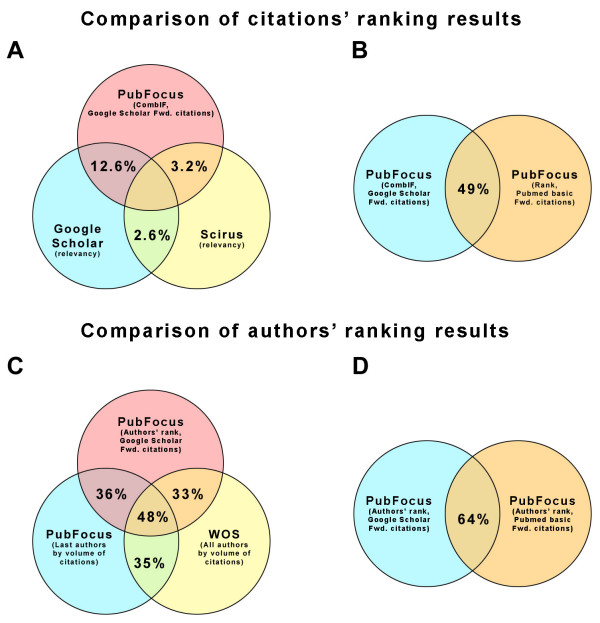
**Comparison of ranking results between search engines**. **A) **Low degree of the overlap between PubFocus, Google™ Scholar and Scirus™ for the top 10 ranked citations. PubFocus (with Google™ Scholar as the source of forward citations) and Google™ Scholar had 12.6% of common citations. PubFocus (with Google™ Scholar as the source of forward citations) and Scirus™ had only 3.2% of common citations, and Google™ Scholar and Scirus™ had only 2.6% of citations in common. **B) **49% of identical top 10 ranked citations were returned by PubFocus when Google™ Scholar and PubMed Basic sources of forward citations were used as alternative. **C) **Partial overlap of the top 10 ranked authors between PubFocus and Web of Science^®^. PubFocus (based on Author's rank with Google™ Scholar as the source of forward citations) and Web of Science^® ^(volumetric statistics on all authors) had 33% of common authors. 36% of authors were the same between two PubFocus outputs based on Author's rank and volumetric data. Volumetric Web of Science^® ^and volumetric PubFocus results had 35% of common authors. **D) **64% of identical top 10 authors were returned by PubFocus when Google™ Scholar or PubMed Basic sources of forward citations were used in Authors' rank calculation.

We compared the degree of overlap of top 10 search results between PubFocus, Google™ Scholar and Scirus™ for 50 different search queries (i.e. a total of 500 biomedical articles from each search engine). Each search query was designed to cover a separate biomedical topic and to have no or minimal degree of overlap with the rest of the search queries [see Additional file 1]. contains the definition of each search query used and raw search output data. Presented search queries are in the PubMed format. Queries' syntax was changed accordingly to comply with Google™ Scholar and Scirus™ formats. Comparison of the top 10 search results revealed a low degree of overlap between the 3 search engines (Fig. 4A). PubFocus (with Google™ Scholar as the source of forward citations) and Google™ Scholar had 12.6% of citations in common. PubFocus (with Google™ Scholar as the source of forward citations) and Scirus™ had only 3.2% citations in common, and Google™ Scholar and Scirus™ had only 2.6% of citations in common. 49% of identical top 10 citations were returned by PubFocus when Google™ Scholar and PubMed Basic served as alternative sources for forward citations (Fig. 4B). This demonstrates the significance of a more quantitative source of forward citations (Google™ Scholar) on the outcome of ranking.

**Figure 5:**
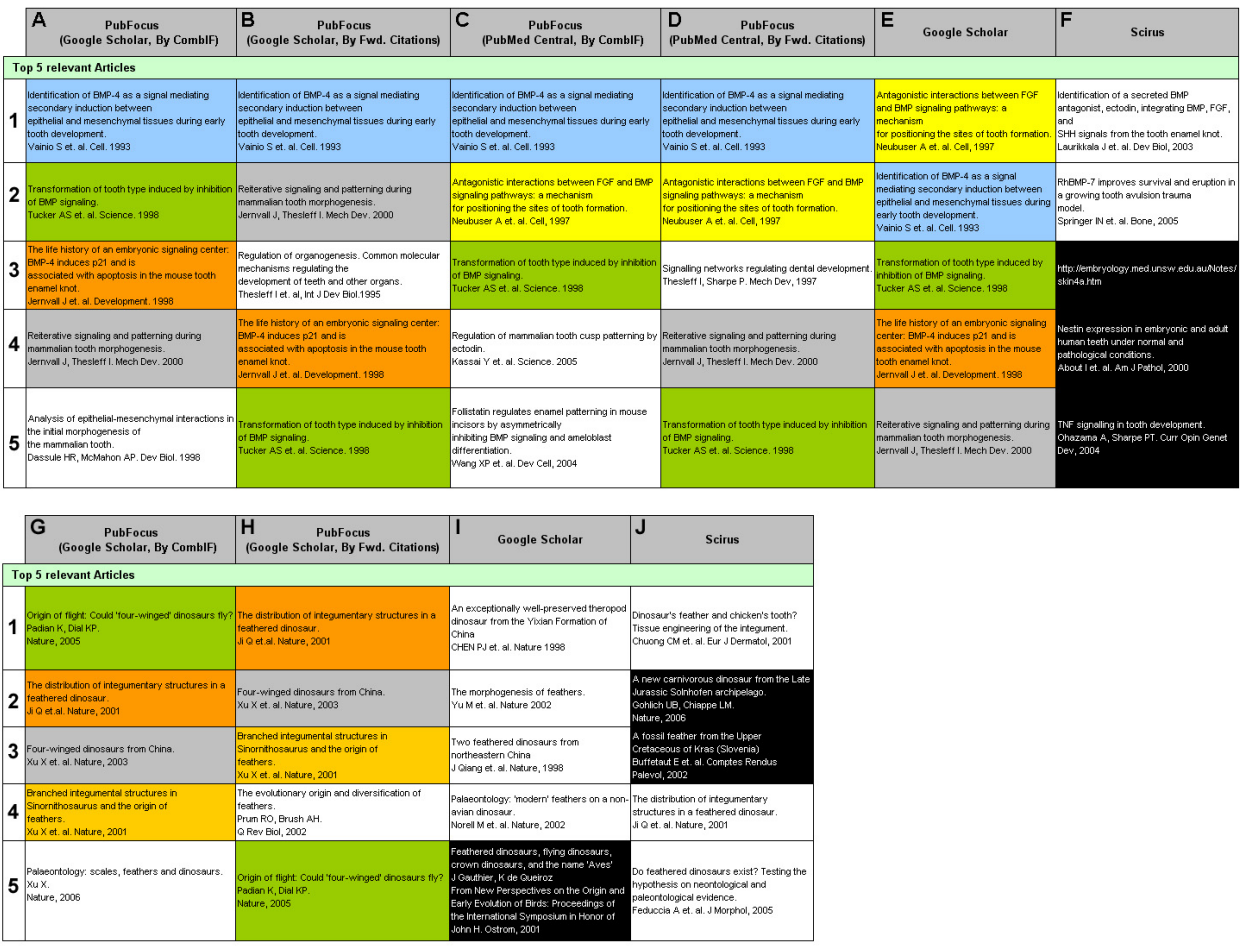
**Comparison of top 5 search results, example queries**. Comparison of top 5 search results for the search query "*(BMP) AND (tooth OR teeth) NOT (hair OR beak)*" (A-F) and for the search query "*dinosaur feather*" (G-J) based on their relevancy between PubFocus, Google™ Scholar and Scirus™ services. For each record title, authors, publication source and publication year are listed. Identical records between columns have the same colour coding. Records presented in white-on-black mode were subjectively estimated to have either low relevancy to the original search query or to originate from the source with arguable scientific credibility. **A, G) **Top 5 PubFocus results based on Publication Impact Factor with Google™ Scholar used as the source of forward citations. **B, H) **Top 5 PubFocus results based on Google™ Scholar forward citations only. **C) **Top 5 PubFocus results based on Publication Impact Factor with PubMed Central used as the source of forward citations. **D) **Top 5 PubFocus results based on PubMed Central forward citations only. **E, I) **Top 5 Google™ Scholar results. **F, J) **Top 5 Scirus™ results.

We found variable degrees of cross-search engine overlap of top prioritized results for different search queries. For example, the search query "*(BMP) AND (tooth OR teeth) NOT (hair OR beak)*" should return articles describing role of BMP (Bone Morphogenic Protein) in tooth formation (Fig. 5). There is a high degree of overlap between the top 5 PubFocus results based on either CombIF (Fig. 5A1– A5; Fig. 5C1– C5) or forward citations (Fig. 5B1– B5; Fig. 5D1– D5) and Google™ Scholar results (Fig. 5E1– E5). However, the top five Scirus™ results do not match with either PubFocus or Google™ Scholar (Fig. 5F1– F5). The search query "*dinosaur feather*" should return articles describing evolution of the feathers in dinosaurs. While top 5 PubFocus results based on either CombIF (Fig. 4G1– G5) or Google™ Scholar forward citations overlap (4 out of 5 articles are the same; Fig. 4H1– H5), there is no overlap with Google™ Scholar (Fig. 4I1– I5) or Scirus™ results (Fig.4J1– J5).

### Comparison of statistical functionality between PubFocus and Web of Science^®^

Both PubFocus and Web of Science^® ^offer statistical tools to sort and rank search results by various parameters (Fig. 3). However, Web of Science^® ^outputs statistical data based on volumetric parameters only. This is not ideal and can be misleading. For example, volumetric ranking of authors in Web of Science^® ^does not account for the impact of their publications or the author's role in the project and does not provide an accurate representation the leading scientists in the field. PubFocus accounts for the above-mentioned parameters and its ranking of authors can be more reliable and more insightful.

**Figure 6:**
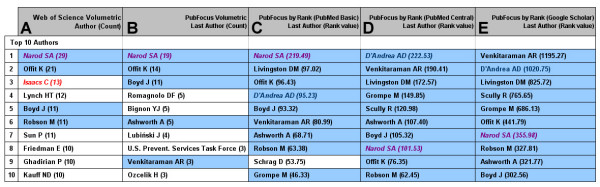
**Author ranking strategies**. Author ranking strategies for the articles returned by searching for "BRCA". Author's names matching between columns are colour coded with blue background. Italicized and coloured author's names are subjectively evaluated in details within Results. **A) **Top 10 authors by volume produced by Web of Science^®^. **B) **Top 10 last authors (principal investigators) by volume produced by PubFocus. **C) **Top 10 most impacting authors by AR (Author's Rank) produced by PubFocus (Forward citations were collected in "PubMed Basic" mode). **D) **Top 10 most impacting authors by AR produced by PubFocus (Forward citations were collected in "PubMed Central" mode). **E) **Top 10 most impacting authors by AR produced by PubFocus (Forward citations were collected in "Google™ Scholar" mode).

Consider a search for "BRCA". BRCA1 (BReast-CAncer susceptibility gene 1) and BRCA2 are tumor suppressor genes implicated in DNA repair, transcriptional control after DNA damage and chromosomal stability. Mutation in these genes predispose to breast and ovarian cancer [27]. As of 04/14/2006, this search query has returned 610 PubMed matches encompassing the molecular, genetic, clinical and epidemiological aspects of BRCA genes (analogous search in Web of Science^® ^resulted in 800 matches). We performed comparative analysis of top authors based on volumetric approach in Web of Science^® ^and PubFocus, as well as based on authors' impact ranking derived from Author's Rank (AR; see Implementation). AR was calculated using three alternative sources of forward citations: PubMed Basic, PubMed Central or Google™ Scholar (see Implementation; Fig. 2D). Ranking based on AR in three alternative modes returns highly overlapping lists of top 10 most impacting authors (compare Fig. 6C–6E. 100% overlap between 6D and 6E and 90% overlap with 6C). Volumetric ranking in Web of Science^® ^and PubFocus returns lists of top 10 most publishing authors that only partially overlap with the top 10 most impacting authors (Fig. 6A, 6B).

We performed subjective assessments of the impact of several authors from publications obtained in the above ranking lists. *Narod SA *was identified to be one of the leading scientists in the field of BRCA study by all 5 ranking approaches used. Indeed, *Narod SA *has numerous highly cited publications on various clinical aspects of BRCA-associated cancers with some studies published in high profile journals such as *New England Journal of Medicine (impact factor – 38.57)*. PubFocus ranking has also identified highly influential authors otherwise overlooked by volumetric approaches. For instance, *D'Andrea AD *has 7 highly cited publications on the genetic aspects of BRCA and BRCA involvement in ovarian cancer with several studies published in high profile journals such as *Nature Medicine (impact factor – 31.2)*. However,*D'Andrea AD *was not identified by Web of Science^® ^as a top contributing author. On the other hand, *Isaacs C*, ranked third by Web of Science^®^, was not included in top 10 lists based on ARs in PubFocus. Subjective analysis shows that *Isaacs C *was involved in some publications, among others, that aimed to identify secondary breast cancer risk factors (such as smoking and coffee consumption) in BRCA mutation carriers. In all of these publications, *Isaacs C *does not appear to play leading roles (i.e. in these publications *Isaacs C *is neither first nor last author).

We have further compared the degree of overlap of top 10 authors between PubFocus and Web of Science^® ^for the 50 search queries (same search queries as in the above section were analysed [see Additional file 1]. Syntax of PubMed queries was changed accordingly to comply with Web of Science^® ^formats). Comparison revealed only a partial degree of overlap between 2 search engines (around 1/3; Fig. 4C). PubFocus (based on Author's rank with Google™ Scholar as the source of forward citations) and Web of Science^® ^(volumetric statistics on all authors) had 33% of authors in common. PubFocus (based on Author's rank with Google™ Scholar as the source of forward citations) and PubFocus (volumetric statistics on last authors) had 36% of authors in common. Web of Science^® ^(volumetric statistics on all authors) and PubFocus (volumetric statistics on last authors) had 35% of authors in common. 64% of identical top 10 authors were returned by PubFocus when Google™ Scholar or PubMed Basic sources of forward citations were used in the Authors' rank calculation (Fig. 4D). This again underlines the significance of the more quantitative source of forward citations (Google™ Scholar) on the outcome of ranking.

### Other practical implementations of PubFocus ranking functionality

The statistical and search narrowing tools of PubFocus can be used in a variety of ways apart of those described above to quickly gain important information about the field of search. Here, we will limit ourselves to exemplifying several other aspects of its functionality in another case study. Consider a search for "*(hair follicle) AND (stem cells)*". As of 04/14/2006, this search query has returned 322 PubMed matches encompassing the field of stem cell biology of hair follicles. We can use statistical tools to address following questions:

**1) What are the publication trends in the field and what are the keystone articles? **Quick evaluation identifies continuing expansion of the field that started around 1990 (based on the "*Publications per Year*" graph accessible under the "*Basic Statistics*" tab). Focusing our search on the subset of articles published from 1990 to 1994 (when the field was beginning to expand) and further focusing on the top three journals with the highest impact factors (in this case: *Cell*, *J Cell Biol *and *PNAS*) leaves us with 5 articles. These articles could potentially contain findings that have sparked further field expansion. Indeed, among these 5 articles we find a keystone article by *Cotsarelis *[28] that for the first time correctly identified stem cells to reside in a bulge region of the hair follicle instead of the lower bulb region. This finding was later confirmed in other publications and had a major impact on the field. An alternative method of accessing this keystone article would be first to limit search to articles published from 1990 to 1994 and further to limit search to top three ranked articles based on CombIF (based on the "*Distribution of Combined Impact Factor Values*" graph accessible under the "*Basic Statistics*" tab).

**2) What are the preferable journals for publications in the field? **This can be an important question to be answered for prospective authors looking to publish their article on the relevant topic in the most appropriate source. By looking at the distribution of "*Top 10 Publishing Journals*" (this functionality is similar to that offered by Web of Science^®^) for the field of hair follicle stem cells we can clearly see that most of the articles were published in the *Journal of Investigative Dermatology*. Furthermore, PubFocus generates a distribution of "*Top 10 Publishing Journals by Impact Factor*" where highest profile journals are identified first. This graph allows identification of sources where most of the important papers on the topic are published (*Cell, Science, Nature, Nature Medicine *etc). This graph can also be used as an alternative starting point to the "*Distribution of Combined Impact Factor Values" graph in a search narrowing strategy aim*ing to focus on the most impacting publications first.

**3) What does an author's publication profile look like? **We can further expand our search for a single author (here *Cotsarelis G*.) by accessing the "ONLY" link next to the author's name. Evaluation of publication trends by *Cotsarelis G *identifies a continuous positive trend (out of 45 matches as of 04/14/2006). Focusing on articles published in journals with the highest impact factor (in this case: *N Engl J Med, Science, Nature Medicine, Cell*), we are left with six highly ranked articles by this author (again, an alternative strategy of focusing on top ranked articles from the "*Distribution of Combined Impact Factor Values*" graph can be used).

By evaluating articles by principal investigators with whom *Cotsarelis *has co-authorship, we can identify other scientists *Cotsarelis *collaborates with. In this case, *Cotsarelis *appears to closely collaborate with *Lavker R.M*. and *Sun T.T*. However, further focusing the search on these two authors reveals that *Cotsarelis *had last co-authorship with them in 1999. In following years, *Cotsarelis *predominantly worked as the principal investigator on his own projects.

A different trend can be observed for another established scientist in the field of hair biology, *Sundberg J.P*. A search for "*Sundberg JP*" as of 04/14/2006 returns 258 matches spanning from 1977 to present. By further focusing on the work of this author with other principal investigators such as *Hoffmann R., Christiano A.M*., and *Paus R*., we find collaborative efforts on 19 articles from 1998 to 2005.

### Integration of biomedical terms extraction into the semantic search in PubFocus

Controlled dictionaries and ontologies (here, NCI thesaurus or MGD mammalian gene orthology database) can be used to generate textual reference points composed of relevant biomedical terms. These can be combined with the bibliomertic reference points described above to form an efficient Pubmed citations searching strategy. This strategy requires minimal prior knowledge of the field of search and can be successfully used by these who have a basic understanding of relevant biomedical terminology. The exact sequence of the use of reference points is subject to variability and depends on the goals of search. Here we will limit ourselves to just a few examples outlining the basics of this data mining process.

**1) Molecular basis of somites segmentation**. Query 3: *(somite [ti] or somites [ti])AND(segmentation) *inquires about the process of somite segmentation and returns 115 citations [see Additional file 1]. In the first step we will look at the genes involved in the process of segmentation by accessing the "Gene Orthology" tab. Now we can focus on the genes from signalling pathways with oscillatory behaviour (so-called "clock" genes): *HES1, HES5, HES6, HES7, NOTCH1, NOTCH2, DLL1 LFNG, WNT3A, DVL1 *and *DVL2*, as well as homeobox-containing proteins: *PITX2*, *TBX18 *and *TBX6*. Focusing on these genes narrows our search to 19 citations. In the second step we will focus our search on model organisms to filter out invertebrate organisms by accessing the "NCI Ontology tab" and "Organisms" menu option. We select all options but: DROSOPHILA and INVERTEBRATE. This step narrows our search to 8 articles. In the third step, we will look at the relevant "Properties" terminology from the NCI database. Particularly, we will select terms relevant to the polarity and symmetry: POSTERIOR, ANTERIOR, POLARITY, LATERAL, DISTAL and PROXIMAL. This step brings out 5 relevant articles. In the fourth step, we will go to the "Statistics: Top 10" tab and will select 2 articles with the highest CombIF values. Now we can access the "Sorted results" tab to review our selection.

**2) Cancer and inhibitors of heat shock protein 90**. Query 4: (Hsp90 inhibitor* [ALL]) inquires about heat shock protein 90 inhibitors and returns 286 citations [see Additional file 1]. In the first step, we will look at the associated malignant conditions by accessing the "NCI Ontology" tab and "Diseases" menu option. We select for: LEUKEMIA, LYMPHOMA, CARCINOMA, GLIOMA, LEUKEMIAS, MYELOMA, MALIGNANCY, SARCOMA, MELANOMA, SCLC, NEOPLASM, NEOPLASIA, NEOPLASM, NEOPLASMS, OSTEOSARCOMA, RETINOBLASTOMA, HEPATOMA. This step narrows our search to 51 articles. In the second step, we will look at the "Anatomical Terms" from the NCI database. We will select our organ of interest – PROSTATE. This step brings out 6 relevant articles. In the third step, we will go to the "PI Authors" tab and will select: SHERMAN MY as the highest ranked author. Now we can access "Sorted results" tab to view the selected publication.

**2) Sensory systems in Platypus**. Query 47: *(platypus [ALL]) *inquires about research on the unique monotreme mammal and returns 298 citations [see Additional file 1]. In the first step we will look at the associated neural and sensory structures in Platypus by accessing the "NCI Ontology tab" and "Anatomical Terms" menu option. We select for: MECHANORECEPTOR, MECHANORECEPTORS, CORTEX, AXON, GANGLION, GANGLIA, ASTROCYTE, ASTROCYTES, DENDRITE, MYELIN, NEUROFILAMENT, PHOTORECEPTOR, PHOTORECEPTORS, OLIGODENDROCYTE, OLIGODENDROCYTES, THERMORECEPTOR, THERMORECEPTORS. This step narrows our search to 38 articles. In the second step we will go to the "PI Authors" tab and will select for top 5 ranked authors for this topic: PROSKE U, IGGO A, ASHWELL KW, PETTIGREW JD, KRUBITZER L. This step narrows our search down to 17 articles. In the third step, we will look at "Activities" terminology from the NCI database. We will select for: DETECTION, ANALYSIS and PROCESSING. This step brings out 6 relevant articles. Now we can access "Sorted results" tab to view selected publications.

## Discussion

Compatibility of knowledge representation with human conceptual understanding determines usability of the knowledge source, simplicity and speed of the knowledge acquisition process [29]. Fast-pace growth of biomedical knowledge calls for the development of new, efficient information representation and decision support tools. MEDLINE/PubMed is one of the most important biomedical databases serving as an every-day reference point for scientists, physicians and other health-care professionals. Knowledge in PubMed is arranged in the form of a rectangular database, where each entry (i.e. citation) is assigned with the same attributes. While this makes the PubMed database compatible with standard data sorting and representation operations, it imposes limitations on the intuitive accessibility of information. An individual working with PubMed typically has to read through chronologically arranged citation records (titles, abstracts or full length articles) and base his or her judgment on the significance of the article from personal knowledge of the subject (such as knowledge of appropriate terminology). Consequently, this can be inaccurate, as one might not have enough knowledge and experience to make an objective determination of an article's significance. Additionally, this strategy may also be laborious and time-consuming. A semantic network is a more intuitive and progressive data representation method [29]. Semantic networks can be built around the PubMed database where each citation record is connected with multiple entries from other biomedical sources and databases. In turn, these relevant entries can serve as insightful reference points in computer-assisted PubMed citations analytics. Development of efficient semantic networking tools for publications analytics is an important concern in biomedical informatics.

We see two major challenges on the path to building efficient semantic networks for PubMed database analytics. The first challenge pertains to the development of meaningful, interest-specific and extensive extraction and representation of biomedical terms. The second relates to the development of reliable algorithms for sorting and ranking citations. We believe that construction of successful semantic networks for PubMed would include development of automated terminology extraction tools based on existing biomedical thesauri, dictionaries and ontologies, and ranking algorithms based on established "human factor"-based bibliometric parameters such as journal impact factor and forward references.

To address these challenges we have built a scalable prototype PubMed citation analytics system (PubFocus). The following three steps of data mining were realised in this system: 1) Query match-based selection of the records from PubMed database (for this step PubFocus relies on the build-in PubMed search engine); 2) Controlled vocabularies-assisted extraction of relevant biomedical terms (NCI thesaurus and MGD mammalian gene orthology database were implemented for this project); 3) prioritization of the resulting citations based on the algorithm relying on journal impact factor, volume of forward references, referencing dynamics and authors' contribution level. For each citation PubFocus generates multiple reference points including bibliometric (impact factor, forward citations volume, rank) and contextual (matching biomedical terms fro controlled vocabularies, ontologies). Combined and statistically processed, these reference points are used in computer-assisted citation analytics.

Scalability of PubFocus allows expansion of the bank of controlled vocabularies. A single controlled vocabulary or ontology contains a collection of biomedical terms of special interest often useful for the analytics of the limited group of PubMed citations (such as FlyBase – a database of Drosophila melanogaster genome) [30]. As the result of such "specialization" of the terminology, multiple controlled vocabularies covering various biomedical fields should be used simultaneously. Many of these terminology databases have already been built. A majority of them are available within the public domain (as many as 2,704 biomedical databases have been made available through BioMed Central) [11].

PubFocus can be adapted to work with citation collections other than PubMed. Properly formatted in MySQL, a variety of databases can be readily queried by PubFocus. For example, PubFocus analytics can be integrated into publisher's databases, biomedical journals, university libraries, R&D databases of commercial organisations etc.

Several challenges remain on the path toward optimisation of the efficient semantic networking tools for publications analytics. Citations ranking algorithms should be further evaluated. Comparison of ranked results between major proprietary ranking algorithms (Google™ Scholar and Scirus™) and between these and PubFocus have uncovered very low degree of overlap. It should be further determined what should and what should not constitute a meaningful factor in the ranking process. Here we propose an algorithm that is largely based on journal impact factor and forward citations volume and dynamics. However, other "human factor"-based parameters can be used as well. Each PubMed citation can be assigned with the rank based on experts' opinion. It can be generated upon formal post-publication evaluation performed by other scientists working within the relevant field [13]. However, for obvious reasons it is difficult to generate such parameters for all the articles in the PubMed database. An effort has been initiated by Biomed Central toward this direction with the introduction of "Faculty of 1000 (F1000) Biology" and "F1000 Medicine". Both new services attempt to "comprehensively and systematically highlight and review the most interesting papers published in the biological sciences based on the recommendations of a faculty of well over 1000 selected leading researchers" and "in any field of medicine based on the recommendations of a selected faculty of nearly 2500 leading international researchers and clinicians" [31,32]. If successful, F1000s can deliver a new citation ranking parameter.

Further integration of PubFocus with the available controlled dictionaries and ontologies is under way. A robust platform for categorizing, evoking and switching between these dictionaries needs to be built. Additionally, integration of PubFocus metasearch into alternative citations databases, such as university libraries or biomedical journal databases will be conducted. Similar efforts have been taken by commercial organizations. For example, Vivisimo^® ^metasearch "automatically categorizes search outputs into hierarchical clusters" and has been integrated into the PNAS (Proceedings of the National Academy of Sciences) journal database and the University of Pittsburgh search engines.

## Conclusion

We have built a prototype semantic networking tool for the PubMed citations analytics. It performs automated biomedical terminology extraction based on the controlled dictionaries and publications ranking based on two available but previously uncoupled human factor-based parameters: journal impact factor and forward citations.

## Availability and requirements

**Project name: **PubFocus

**Project home page: **

**Operating system(s): **Linux/Unix

**Programming language: **PERL

**Other requirements: **MySQL database. Perl modules for XML processing, HTML parsing, generation of session, fork processes and interaction with MySQL database.

**License: **Open Access.

**Any restrictions to use by non-academics: **n/a.

## Authors' contributions

MVP developed software, carried out testing and drafted the manuscript. ZZ conceived of the study, and participated in its design and coordination and helped to draft the manuscript. CMC participated in the evaluation of the software and the manuscript.

## Supplementary Material

Additional file 1 Comparison of search results PubFocus, Web of Science^®^, Google™ Scholar and Scirus™. This file contains the definition of 50 search queries used in this study and raw search output data from PubFocus, Web of Science^®^, Google™ Scholar and Scirus™. Click here for file
